# Predictive value of neutrophil-to-lymphocyte ratio, platelet-to-lymphocyte ratio, and monocyte-to-lymphocyte ratio for three-year survival in patients with early esophageal cancer undergoing endoscopic submucosal dissection

**DOI:** 10.3389/fonc.2025.1714984

**Published:** 2026-01-07

**Authors:** Yanfang Zheng, Hong Zhang, Yuchen Su, Jie Zhang, Wei Xu, Weihao Pan, Zhigang Li

**Affiliations:** 1 Department of Esophagoscopy Room, Shanghai Chest Hospital, Shanghai Jiao Tong University School Of Medicine, Shanghai, China; 2 Department of Thoracic Surgery, Shanghai Chest Hospital, Shanghai Jiao Tong University School Of Medicine, Shanghai, China

**Keywords:** early esophageal cancer, endoscopic submucosal dissection (ESD), interaction, monocyte-to-lymphocyte ratio (MLR), neutrophil-to-lymphocyte ratio (NLR), platelet-to-lymphocyte ratio (PLR)

## Abstract

**Background:**

Endoscopic submucosal dissection (ESD) has been widely applied as an effective treatment for early esophageal cancer. However, long-term prognosis after surgery remains highly variable. This study aimed to evaluate the clinical value of NLR, PLR, and MLR in patients with early esophageal cancer treated with ESD.

**Methods:**

A total of 325 patients with early esophageal cancer who underwent ESD were retrospectively included and categorized into survival and death groups based on their three-year postoperative outcomes. Baseline characteristics between groups were compared using univariate analysis. The Cox proportional hazards model was applied to assess the associations of NLR, PLR, MLR, and their interactions with three-year survival. Restricted cubic spline (RCS) regression was performed to explore the potential nonlinear relationships between NLR, PLR, MLR, and survival outcomes. Receiver operating characteristic (ROC) curves were used to evaluate the predictive ability of NLR, PLR, and MLR for three-year survival.

**Results:**

Baseline characteristics were significantly better in the survival group compared with the death group. The Cox proportional hazards model revealed that higher discharge levels of NLR, PLR, and MLR were significantly associated with increased risk of three-year mortality, with notable synergistic effects observed with tumor location, depth of invasion, tumor size, and margin status. RCS analysis demonstrated significant linear or near-linear associations between NLR, PLR, MLR, and mortality risk. ROC curve analysis indicated that NLR, PLR, and MLR exhibited good predictive performance for three-year survival, with PLR showing superior predictive ability.

**Conclusion:**

NLR, PLR, and MLR are significantly and positively associated with three-year mortality risk in patients with early esophageal cancer undergoing ESD, in either linear or near-linear patterns. These markers also exhibit synergistic interactions with tumor-related clinical features such as location, depth of invasion, and margin status. Among them, PLR demonstrated the best predictive performance. This study provides valuable evidence for postoperative risk assessment and individualized follow-up management in early esophageal cancer patients.

## 1 Introduction

Esophageal cancer is one of the most common malignant tumors of the digestive tract worldwide (1, 2) with high incidence and mortality rates (3), seriously threatening patients’ life and health. Although screening techniques and diagnostic and therapeutic levels have improved in recent years, allowing some patients to be detected at an early stage, the overall prognosis of esophageal cancer remains unsatisfactory (4). Early esophageal cancer, since distant metastasis has not yet occurred, has a relatively better postoperative prognosis and a higher cure rate (5). However, even after undergoing endoscopic submucosal dissection (ESD) treatment (6, 7), patients still exhibit considerable variability in long-term outcomes. While some patients experience favorable prognoses, others develop tumor progression or even die within several years following ESD. Therefore, accurately identifying high-risk individuals at an early postoperative stage has become a critical issue in clinical management. Currently used prognostic indicators—such as depth of invasion and resection margin status—are difficult to obtain and are derived from invasive diagnostic procedures (8, 9). Hence, there is an urgent need to develop easily accessible and reliable biomarkers to improve the assessment of long-term survival risk after ESD.

In recent years, the role of inflammatory response in the occurrence, development, and metastasis of tumors has received widespread attention. Chronic low-grade inflammation can promote tumor cell proliferation, inhibit apoptosis, and alter the tumor microenvironment to facilitate tumor invasion and metastasis through multiple mechanisms. At the same time, inflammatory cells and their secreted cytokines, chemokines, and growth factors can promote angiogenesis, immune escape, and tumor drug resistance. At present, many studies have shown that peripheral blood parameters neutrophil-to-lymphocyte ratio (NLR) (10–12), platelet-to-lymphocyte ratio (PLR) (13, 14) and monocyte-to-lymphocyte ratio (MLR) (15, 16), can serve as prognostic indicators for various cancers, assessing patients’ postoperative survival or the risk of disease recurrence. However, for patients with early esophageal cancer undergoing endoscopic mucosal resection (EMR), studies on the relationship between peripheral blood parameters and postoperative prognosis are still limited, and the existing evidence is insufficient. Additionally, the potential impact of the interactions between these peripheral blood parameters and other clinical features on postoperative prognosis has yet to be fully explored. Therefore, this study aims to evaluate the prognostic value of NLR, PLR, and MLR in patients with early esophageal cancer undergoing ESD, providing new biomarker evidence for identifying high-risk patients after surgery and for individualized management.

## 2 Materials and methods

### 2.1 Inclusion and exclusion criteria

This study retrospectively included 325 patients with early esophageal cancer who underwent ESD at our hospital from June 2018 to December 2024. The inclusion criteria were: 1) age ≥18 years; 2) clinically and pathologically confirmed early esophageal cancer (T1a or T1b stage); 3) meeting the indications for endoscopic submucosal dissection (ESD). The exclusion criteria were: 1) concomitant severe systemic diseases, such as other malignancies or major immune deficiencies; 2) severe liver or kidney dysfunction; 3) tumor invasion into the muscularis propria; 4) receiving chemotherapy or other treatments prior to enrollment; 5) pregnant or breastfeeding women; 6) missing essential data. We used a consecutive enrollment approach, initially including a total of 387 patients who underwent esophageal ESD. After applying the inclusion and exclusion criteria, 325 patients were ultimately included in the analysis. All patients were scheduled for a 3-year follow-up, which was conducted via outpatient visits and telephone calls. The 3-year follow-up completion rate was 87.4%. The detailed patient selection flowchart is shown in **Supplementary Figure 1**. A graphical summary of the study design is provided in **Supplementary Figure 2**.

### 2.2 Surgical procedure

Patients underwent ESD under general anesthesia. After endotracheal intubation, endoscopic examination was performed. Narrow-band imaging (NBI) and Lugol’s iodine staining were used to delineate the lesion boundaries. Marks were made at the edges of the lesion, and dye was injected at the marked sites. After adequate lesion lifting, an incision was made 5 mm outside the marks using a needle knife (Olympus Dual knife). The submucosal layer was incised, and the lesion mucosa was dissected. The submucosal tissue was gently pushed aside to expose the operative field and stretch the lesion tissue, ensuring complete resection. Electrocoagulation was applied for hemostasis. For patients with deeper submucosal dissection, titanium clips were used to close the wound, and gastric mucosal protectants were sprayed to promote healing.

### 2.3 Data collection

The collected data mainly included general demographic information, tumor-related information, surgical and hospitalization details, as well as peripheral blood and inflammatory markers.

General demographic information: age, gender, body mass index (BMI), smoking and drinking history, and comorbidities including hypertension, diabetes, and coronary heart disease.

Tumor-related information: lesion location (distance from incisors, cm), tumor volume (mL), pathological type (squamous cell carcinoma, adenocarcinoma, or neuroendocrine carcinoma), resection margin status (negative or positive), histological differentiation grade (well, moderate, or poor), depth of mucosal or submucosal invasion (T1a or T1b), and presence of lymphovascular invasion.

Surgical and hospitalization details: duration of surgery (minutes), intraoperative blood loss (mL), and length of hospital stay (days).

Peripheral blood and inflammatory marker: white blood cell count (WBC, 10^9^/L), neutrophil count (Neutrophils, 10^9^/L), lymphocyte count (Lymphocyte count, 10^9^/L), monocyte count (Monocyte count, 10^9^/L), eosinophil count (Eosinophils, 10^9^/L), basophil count (Basophils, 10^9^/L), platelet count (Platelet count, 10^9^/L), red cell distribution width (RDW, %), and inflammation-related ratios, including neutrophil-to-lymphocyte ratio (NLR), platelet-to-lymphocyte ratio (PLR), and monocyte-to-lymphocyte ratio (MLR), all collected at the time of patient discharge.

### 2.4 Statistical analysis

All data were analyzed using R software (version 4.4.1). Continuous variables were expressed as median (minimum–maximum) and compared between groups using the Mann–Whitney U test; categorical variables were expressed as counts (percentages) and compared using the Chi-square test or Fisher’s exact test. Postoperative 3-year survival status (Alive/Dead) was treated as the dependent variable. Cox proportional hazards models were used to analyze the relationship between peripheral blood markers (NLR, PLR, MLR) and patient mortality risk. NLR, PLR, and MLR were analyzed both as continuous variables and as categorical variables by dividing into quartiles. Interactions between these markers and baseline variables that were significant in univariate analysis were also assessed for their effects on mortality risk. For interaction analyses, continuous variables were dichotomized at the median, and all risk factors were coded as 1 while protective factors were coded as 0 to facilitate interpretation of interaction effects. To evaluate potential non-linear associations, restricted cubic spline (RCS) analyses were performed to examine the dose–response relationship between NLR, PLR, MLR, and mortality risk, with three knots specified; both linear and non-linear trends were tested. Additionally, receiver operating characteristic (ROC) curves were used to assess the predictive ability of NLR, PLR, and MLR for 3-year postoperative survival, with the optimal cut-off point determined as the point corresponding to the maximum Youden index, and sensitivity and specificity were calculated to guide clinical risk stratification. All tests were two-sided, and P < 0.05 was considered statistically significant. For missing data handling, if the outcome variable (such as survival status) is missing or the proportion of missing data exceeds 30%, the corresponding cases will be excluded. For a small amount of missing data (< 5%), median imputation will be used; for missing data between 5% and 30%, multiple imputation will be applied.

## 3 Results

### 3.1 Analysis of intergroup differences between patients alive and dead within three years

#### 3.1.1 Demographic and clinicopathological characteristics

Among the 325 patients, 295 survived three years after ESD, with a survival rate of 90.8%. Compared with the survival group, patients in the death group had significantly higher rates of hypertension (56.7% *vs*. 33.2%, P = 0.018), larger tumor volume (2.1 mL *vs*. 1.6 mL, P = 0.0285), and higher positive resection margin rate (46.7% *vs*. 16.3%, P < 0.001). Conversely, lesion location (distance from incisors: 21.4 cm *vs*. 26.3 cm, P = 0.0019), well-differentiated tumor proportion (16.7% *vs*. 49.8%, P < 0.001), and T1a invasion proportion (43.3% *vs*. 83.7%, P < 0.001) were significantly lower in the death group. Other variables, such as age, gender, body mass index, smoking, and drinking status, did not differ significantly between groups (**Table 1**).

**Table 1:** Baseline characteristics of patients with survival and death within three years.

Characteristic	All patients (n=325)	Alive (n=295)	Dead (n=30)	P-value
Age	68 (25-84)	68 (25-84)	72 (51-83)	0.353
Gender				0.1674178
Male	255 (78.46%)	228 (77.29%)	27 (90%)	
Female	70 (21.54%)	67 (22.71%)	3 (10%)	
BMI	25.2 (19.5-32.7)	25.2 (19.5-32.7)	26.9 (19.7-32.7)	0.333
Smoking				0.1396641
Yes	148 (45.54%)	130 (44.07%)	18 (60%)	
No	177 (54.46%)	165 (55.93%)	12 (40%)	
Drinking				0.213832
Yes	123 (37.85%)	108 (36.61%)	15 (50%)	
No	202 (62.15%)	187 (63.39%)	15 (50%)	
Hypertension				0.01835512
Yes	115 (35.38%)	98 (33.22%)	17 (56.67%)	
No	210 (64.62%)	197 (66.78%)	13 (43.33%)	
Diabetes				0.05773348
Yes	29 (8.92%)	23 (7.8%)	6 (20%)	
No	296 (91.08%)	272 (92.2%)	24 (80%)	
Coronary Heart Disease				0.2596914
Yes	7 (2.15%)	5 (1.69%)	2 (6.67%)	
No	318 (97.85%)	290 (98.31%)	28 (93.33%)	
Lesion location (cm)	25.9 (16.0-38.0)	26.3 (16.0-38.0)	21.4 (16.4-36.7)	0.00188
Tumor size (mL)	1.7 (0.5-2.8)	1.6 (0.5-2.8)	2.1 (0.6-2.8)	0.0285
Histological type				0.1375875
Squamous Cell Carcinoma	310 (95.38%)	283 (95.93%)	27 (90%)	
Adenocarcinoma	12 (3.69%)	9 (3.05%)	3 (10%)	
Neuroendocrine Carcinoma	3 (0.92%)	3 (1.02%)	0 (0%)	
Resection margin status				0.00014881
Negative	263 (80.92%)	247 (83.73%)	16 (53.33%)	
Positive	62 (19.08%)	48 (16.27%)	14 (46.67%)	
Tumor differentiation grade				5.08E-06
Poorly differentiated	72 (22.15%)	55 (18.64%)	17 (56.67%)	
Moderately differentiated	101 (31.08%)	93 (31.53%)	8 (26.67%)	
Well differentiated	152 (46.77%)	147 (49.83%)	5 (16.67%)	
Depth of submucosal invasion				4.90E-07
Mucosa, T1a	260 (80%)	247 (83.73%)	13 (43.33%)	
Submucosa, T1b	65 (20%)	48 (16.27%)	17 (56.67%)	
Presence of lymphovascular invasion				1.21E-06
Yes	32 (9.85%)	21 (7.12%)	11 (36.67%)	
No	293 (90.15%)	274 (92.88%)	19 (63.33%)	
Length of hospital stay (days)	7 (2-36)	6 (2-12)	10 (2-36)	0.000645
Duration of surgery (mins)	81 (40-246)	78 (40-128)	102 (48-246)	0.000297
Intraoperative blood loss (mL)	12 (3-50)	11 (3-20)	13 (4-50)	0.348
Adjuvant therapy				0.8347948
Yes	24 (7.38%)	21 (7.12%)	3 (10%)	
No	301 (92.62%)	274 (92.88%)	27 (90%)	

#### 3.1.2 Peripheral blood parameters at discharge

The results showed that patients in the death group had significantly higher white blood cell counts (9.8 *vs*. 7.7 ×10^9^/L, P = 0.0108), neutrophil counts (8.1 *vs*. 6.5 ×10^9^/L, P = 0.0149), and lymphocyte counts (2.8 *vs*. 2.4 ×10^9^/L, P = 0.0334) compared with the survival group. Furthermore, the neutrophil-to-lymphocyte ratio (NLR, 3.8 *vs*. 2.8, P = 0.00122), platelet-to-lymphocyte ratio (PLR, 357.0 *vs*. 286.2, P = 0.000258), and monocyte-to-lymphocyte ratio (MLR, 0.63 *vs*. 0.47, P = 0.000435) were also significantly elevated in the death group. Platelet counts and red cell distribution width (RDW) did not differ significantly between the two groups (**Table 2**).

**Table 2:** Differences in blood test parameters at discharge between patients with survival and death.

Characteristic	All patients (n=325)	Alive (n=295)	Dead (n=30)	P-value
White Blood Cell, WBC (10^9 cells/L)	7.9 (2.4-12.8)	7.7 (2.4-12.8)	9.8 (3.3-12.8)	0.0108
Neutrophils (×10^9^/L)	6.6 (1.8-11.3)	6.5 (1.8-11.3)	8.1 (2.2-11.2)	0.0149
Lymphocyte count (×10^9^/L)	2.5 (1.3-3.5)	2.4 (1.3-3.5)	2.8 (1.4-3.5)	0.0334
Monocyte count (×10^9^/L)	1.0 (0.3-1.9)	1.0 (0.3-1.9)	1.2 (0.3-1.9)	0.0292
Eosinophils (×10^9^/L)	0.39 (0.05-0.71)	0.37 (0.05-0.71)	0.53 (0.07-0.71)	0.00332
Basophils (×10^9^/L)	0.04 (0.01-0.07)	0.04 (0.01-0.07)	0.05 (0.03-0.07)	0.00157
Platelet count (×10^9^/L)	259.9 (144.2-392.0)	259.9 (144.2-392.0)	259.4 (145.3-384.5)	0.181
Red Cell Distribution Width, RDW (%)	14.1 (11.7-16.4)	14.1 (11.7-16.4)	14.1 (12.0-16.4)	0.822
Neutrophil-to-Lymphocyte Ratio, NLR	2.9 (1.2-4.7)	2.8 (1.2-4.7)	3.8 (1.6-4.7)	0.00122
Platelet-to-Lymphocyte Ratio, PLR	293.3 (142.3-447.7)	286.2 (142.3-447.7)	357.0 (166.4-446.1)	0.000258
Monocyte-to-Lymphocyte Ratio, MLR	0.50 (0.22-0.78)	0.47 (0.22-0.78)	0.63 (0.22-0.74)	0.000435

### 3.2 Effects of inflammatory biomarkers and their interactions on patient survival: Cox proportional hazards analysis

#### 3.2.1 NLR

The results showed that when NLR was analyzed as a continuous variable, it was significantly associated with mortality risk (HR = 1.895, 95% CI 1.290–2.784, P = 0.001), indicating that elevated NLR was related to an increased risk of death. When NLR was categorized into quartiles, compared with the first quartile, the fourth quartile had a significantly higher risk of death (HR = 4.643, 95% CI 1.323–16.293, P = 0.017), while the second and third quartiles showed increased risk (HR = 1.710 and 3.065, respectively) but did not reach statistical significance (P > 0.05). Interaction analysis revealed a significant synergistic effect between NLR and lesion location, with an HR of 2.922 (95% CI 1.227–6.959, P = 0.015), indicating that the combined effect of elevated NLR and lesions closer to the incisors significantly increased the risk of death. Similarly, a significant interaction was observed between NLR and invasion depth, with an HR of 1.927 (95% CI 1.127–3.294, P = 0.017), also greater than 1, suggesting that the association between NLR and mortality risk was more pronounced in patients with deeper tumor invasion (**Table 3**).

**Table 3:** Cox proportional hazards analysis of NLR and interactions on patient survival.

Term	Coef	Std error	Statistic	P value	HR	CI-lower	CI-upper
NLR	0.639	0.196	3.257	0.001	1.895	1.290	2.784
NLR.GroupQ1	ref	ref	ref	ref	ref	ref	ref
NLR.GroupQ2	0.536	0.730	0.734	0.463	1.710	0.409	7.154
NLR.GroupQ3	1.120	0.667	1.680	0.093	3.065	0.830	11.324
NLR.GroupQ4	1.535	0.641	2.397	0.017	4.643	1.323	16.293
NLR*Hypertension	-0.757	0.831	-0.910	0.363	0.469	0.092	2.393
NLR*Lesion location	1.072	0.443	2.422	0.015	2.922	1.227	6.959
NLR*Tumor Size	0.938	0.827	1.134	0.257	2.554	0.505	12.908
NLR*Resection	0.014	0.801	0.018	0.986	1.014	0.211	4.872
NLR*differentiation	-1.350	0.746	-1.810	0.070	0.259	0.060	1.119
NLR*Invasion	0.656	0.274	2.397	0.017	1.927	1.127	3.294
NLR*lymphovascular invasion	0.098	0.838	0.117	0.907	1.103	0.213	5.700
NLR*LOS	-1.351	1.152	-1.173	0.241	0.259	0.027	2.477
NLR*Surgery Time	-1.086	1.157	-0.938	0.348	0.338	0.035	3.263

#### 3.2.2 PLR

The results showed that PLR, when analyzed as a continuous variable, significantly affected survival (HR = 1.009, 95% CI 1.004–1.014, P < 0.001), indicating that elevated PLR was associated with increased mortality risk. When PLR was divided into quartiles, compared with the first quartile, the fourth quartile had a significantly higher risk of death (HR = 7.443, 95% CI 1.691–32.754, P = 0.008), while the second and third quartiles showed increased risk (HR = 3.105 and 4.115, respectively) but did not reach statistical significance (P > 0.05). Interaction analysis indicated a significant synergistic effect between PLR and tumor volume, with an HR of 4.619 (95% CI 1.376–15.507, P = 0.013), suggesting that in patients with larger tumor volume, elevated PLR was significantly associated with increased mortality risk. PLR also showed a significant interaction with resection margin status, with an HR of 1.514 (95% CI 1.013–2.263, P = 0.043), indicating that the association between PLR and mortality risk was stronger in patients with positive resection margins (**Table 4**).

**Table 4:** Cox proportional hazards analysis of PLR and interactions on patient survival.

Term	Coef	Std error	Statistic	P value	HR	CI-lower	CI-upper
PLR	0.009	0.003	3.503	0.000	1.009	1.004	1.014
PLR.GroupQ1	ref	ref	ref	ref	ref	ref	ref
PLR.GroupQ2	1.133	0.816	1.388	0.165	3.105	0.627	15.383
PLR.GroupQ3	1.415	0.791	1.790	0.074	4.115	0.874	19.381
PLR.GroupQ4	2.007	0.756	2.655	0.008	7.443	1.691	32.754
PLR*Hypertension	-0.543	0.958	-0.567	0.571	0.581	0.089	3.798
PLR*Lesion location	0.459	0.986	0.466	0.641	1.583	0.229	10.934
PLR*Tumor Size	1.530	0.618	2.477	0.013	4.619	1.376	15.507
PLR*Resection	0.415	0.205	2.023	0.043	1.514	1.013	2.263
PLR*differentiation	-1.285	0.995	-1.291	0.197	0.277	0.039	1.947
PLR*Invasion	-0.220	0.958	-0.230	0.818	0.803	0.123	5.251
PLR*lymphovascular invasion	-1.180	0.924	-1.277	0.202	0.307	0.050	1.880
PLR*LOS	-0.381	0.976	-0.390	0.696	0.683	0.101	4.625
PLR*surgery Time	0.514	0.986	0.521	0.602	1.672	0.242	11.547

#### 3.2.3 MLR

The results showed that MLR, when analyzed as a continuous variable, significantly affected survival status (HR = 4.255, 95% CI 1.818–9.960, P = 0.001), indicating that elevated MLR was associated with increased mortality risk. When MLR was categorized into quartiles, compared with the first quartile, the fourth quartile had a significantly higher risk of death (HR = 4.144, 95% CI 1.169–14.687, P = 0.028), while the second and third quartiles showed increased risk (HR = 2.379 and 2.706, respectively) but did not reach statistical significance (P > 0.05). Interaction analysis revealed a significant synergistic effect between MLR and lesion location, with an HR of 4.089 (95% CI 1.494–11.192, P = 0.006), suggesting that the association between MLR and mortality risk was stronger in patients with lesions closer to the incisors (**Table 5**).

**Table 5:** Cox proportional hazards analysis of MLR and interactions on patient survival.

Term	Coef	Std error	Statistic	P value	HR	CI-lower	CI-upper
MLR	1.448	0.434	3.338	0.001	4.255	1.818	9.960
MLR.GroupQ1	ref	ref	ref	ref	ref	ref	ref
MLR.GroupQ2	0.867	0.690	1.256	0.209	2.379	0.615	9.201
MLR.GroupQ3	0.995	0.677	1.470	0.141	2.706	0.718	10.199
MLR.GroupQ4	1.422	0.646	2.202	0.028	4.144	1.169	14.687
MLR*Hypertension	0.566	0.916	0.618	0.536	1.762	0.293	10.597
MLR*Lesion location	1.408	0.514	2.741	0.006	4.089	1.494	11.192
MLR*Tumor Size	0.583	1.050	0.556	0.578	1.792	0.229	14.022
MLR*Resection	-0.524	0.914	-0.574	0.566	0.592	0.099	3.549
MLR*differentiation	-0.154	0.660	-0.234	0.815	0.857	0.235	3.124
MLR*Invasion	-0.768	0.958	-0.802	0.423	0.464	0.071	3.034
MLR*lymphovascular invasion	-0.053	0.964	-0.055	0.956	0.948	0.143	6.266
MLR*LOS	-0.217	0.896	-0.243	0.808	0.805	0.139	4.658
MLR*surgery Time	-1.062	1.184	-0.897	0.370	0.346	0.034	3.520

**Figure 1:**
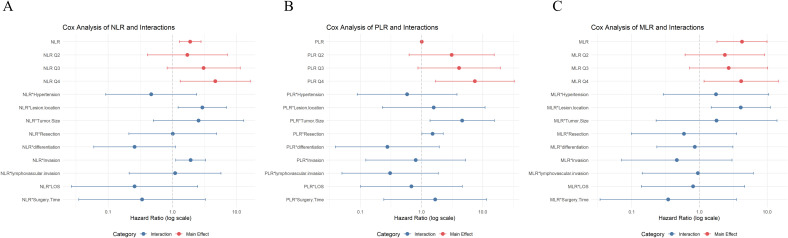
Visual summary of Cox proportional hazards analysis results: **(A)** NLR, **(B)** PLR, **(C)** MLR.

After further adjusting for confounding factors including patient comorbidities and tumor biological characteristics, the three inflammatory markers—PLR, NLR, and MLR—remained independent predictors of survival, with PLR showing the highest significance (**Supplementary Table 1**). This further confirms the robustness of the results reported in our previous analysis. The visual summary of Cox proportional hazards analysis for NLR, PLR, and MLR is presented in **Figures 1A–C**. To account for multiple comparisons, we performed false discovery rate (FDR)-corrected interaction analyses for NLR, PLR, and MLR, and simultaneously tested the proportional hazards assumption using Schoenfeld residuals. The detailed results are presented in **Supplementary Table 2**.

### 3.3 Restricted cubic spline analysis of the relationship between NLR, PLR, MLR and mortality risk

**Figure 2:**
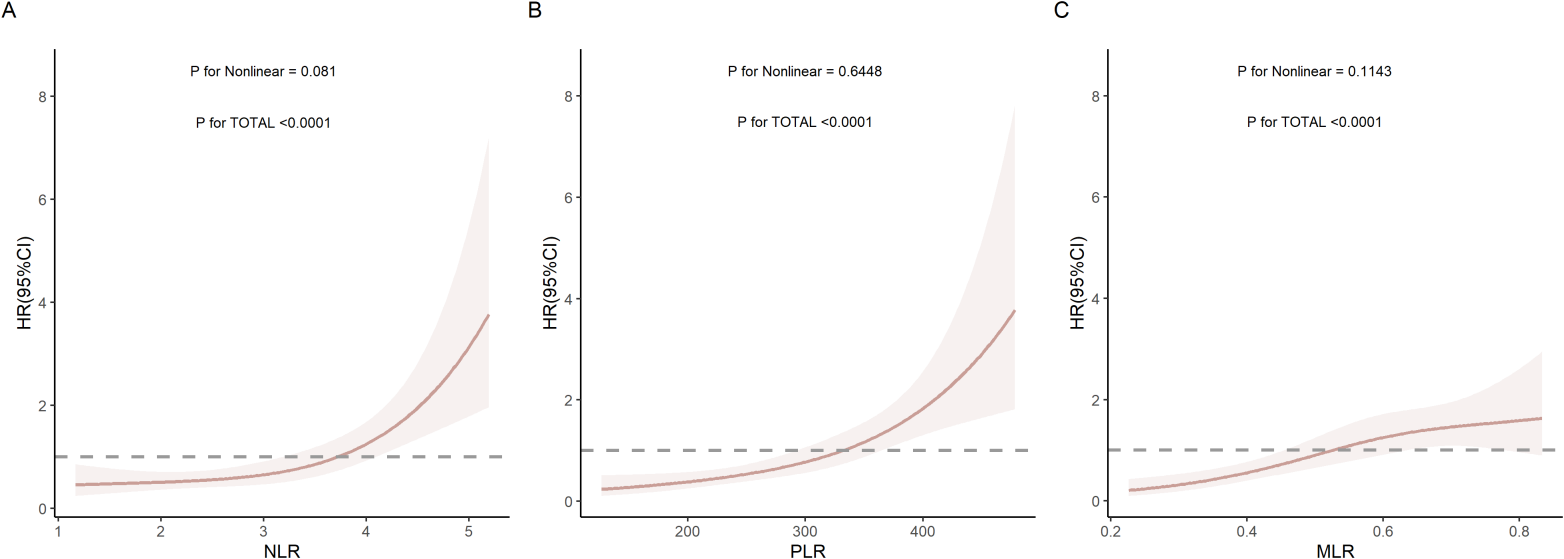
Restricted cubic spline (RCS) curves of **(A)** NLR, **(B)** PLR, and **(C)** MLR with mortality risk.

The results indicated that as NLR, PLR, and MLR increased, patients’ risk of death showed a significant upward trend (P for TOTAL < 0.0001). Linear analysis showed that the P for Nonlinear for NLR, PLR, and MLR were all greater than 0.05, indicating that the non-linear relationship between these three inflammatory markers and mortality risk was not significant. In other words, all three markers exhibited linear or near-linear associations with mortality risk (**Figures 2A–C**).

### 3.4 ROC analysis of the predictive ability of NLR, PLR, and MLR for mortality risk

**Figure 3:**
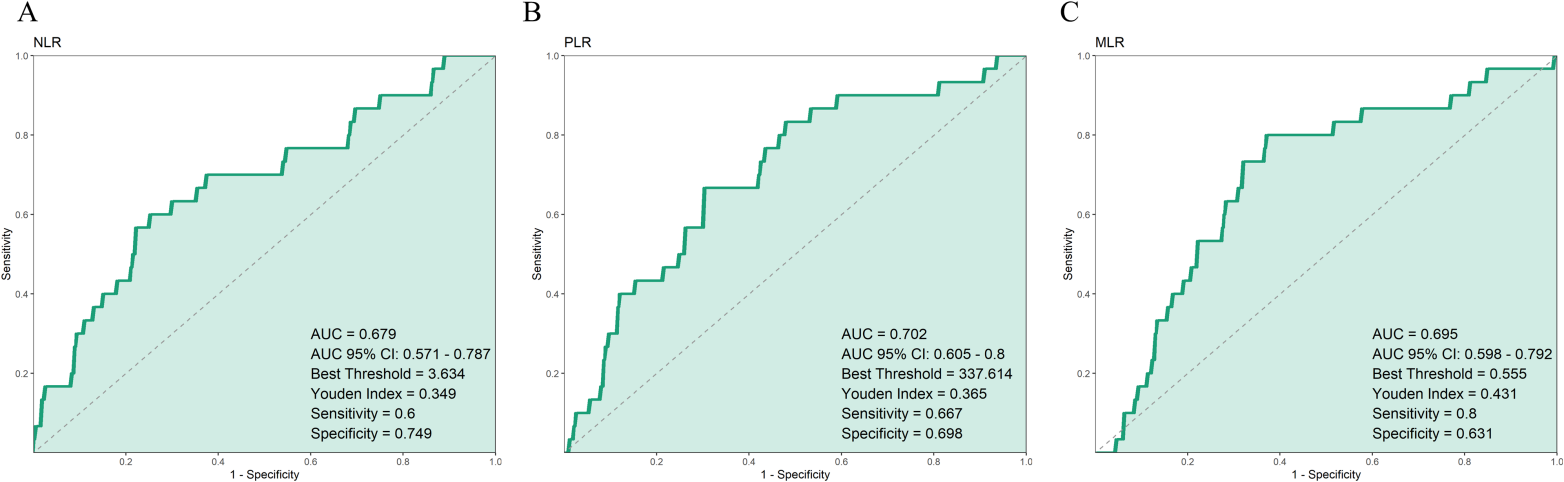
Receiver operating characteristic (ROC) curves of **(A)** NLR, **(B)** PLR, and **(C)** MLR.

The results showed that PLR had the highest area under the curve (AUC = 0.702), followed by MLR and NLR. The optimal cut-off values were 337.6, 0.56, and 3.63, respectively. When exceeding these thresholds, patients had a significantly increased risk of death, indicating that high levels of PLR, MLR, or NLR were associated with poor survival outcomes. Among them, MLR showed the best sensitivity, while NLR had slightly better specificity. All three markers could serve as auxiliary indicators for predicting patient survival status (**Figures 3A–C**).

#### 3.4.1 Sensitivity analysis

**Figure 4:**
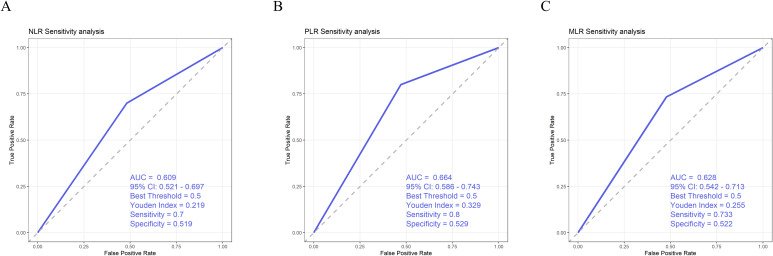
**(A)** Sensitivity analysis of NLR. **(B)** Sensitivity analysis of PLR. **(C)** Sensitivity analysis of MLR.

To further evaluate the robustness of the ROC analysis, we dichotomized NLR, PLR, and MLR based on their median values and re-performed the ROC analyses. The results showed that PLR, NLR, and MLR remained significantly associated with survival outcomes, with PLR exhibiting the highest AUC and superior predictive performance (**Figures 4A–C**). These findings indicate that the conclusions of our study are robust.

### 3.5 Construction of an integrated risk score based on PLR

To further improve the prediction of patient prognosis, we developed a simple integrated risk score model by combining the three most significant clinical features from the multivariable Cox analysis—tumor differentiation, invasion depth, and resection status (all P < 0.001; see **Supplementary Table 1**)—with the best-performing inflammatory marker, PLR. The risk score was calculated using the following formula:

Risk score = 0.005 × PLR + 0.510 × Differentiation + 0.796 × Invasion + 0.612 × Resection.

**Figure 5:**
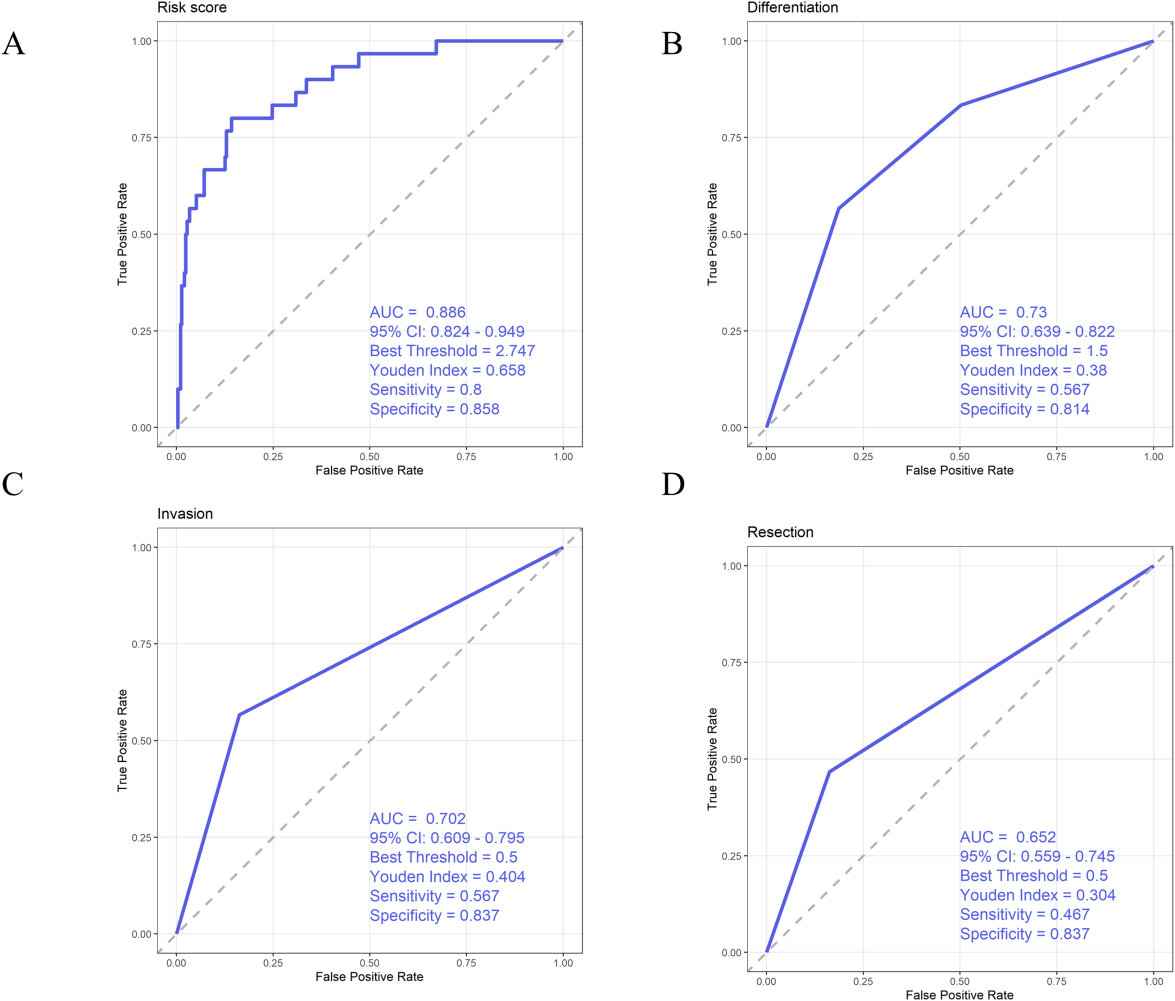
**(A)** ROC curve of the risk score **(B)** ROC curve of tumor differentiation **(C)** ROC curve of invasion depth **(D)** ROC curve of resection status.

The results showed that the risk score achieved the highest AUC for predicting 3-year mortality (**Figure 5**), and DeLong’s test further confirmed that the AUC of the risk model was significantly higher than that of the other four individual indicators (all P < 0.001).

## 4 Discussion

Our study found that patients with higher NLR at discharge had an increased risk of death within three years, which may be closely related to systemic immune status and potential residual lesions. The core reason is that elevated NLR reflects a persistent inflammatory state and systemic immunosuppression. Neutrophils represent innate immunity and possess pro-inflammatory and pro-tumor capabilities (17). They can secrete various cytokines (such as IL-6, IL-8, VEGF, etc.) that promote tumor cell proliferation, angiogenesis, tissue repair (which may also facilitate tumor recurrence), and metastasis. Lymphocytes, on the other hand, represent adaptive immunity and anti-tumor capability, particularly cytotoxic T cells and NK cells, which are the main forces in recognizing and eliminating cancer cells. A high NLR indicates a predominance of neutrophils and relatively fewer lymphocytes, creating a microenvironment that favors tumor growth (18). Although ESD is a radical treatment aimed at complete tumor resection, in some cases, microscopic metastases or minimal residual tumor at the resection margin may not be detectable macroscopically or radiologically. These residual cancer cells continuously release signals, activating systemic inflammatory responses (leading to elevated neutrophils) while simultaneously suppressing effective anti-tumor immunity (resulting in relatively reduced lymphocytes).

Similarly, patients with higher PLR also exhibited an increased risk of death within three years. The core reason is that elevated PLR reflects a platelet-mediated pro-tumor microenvironment and immunosuppression. Circulating tumor cells can activate platelets, causing them to aggregate around the tumor cells and form a physical shield (19). This shield helps tumor cells evade immune surveillance, resist shear stress in the bloodstream, and enhance anchoring and retention in distant vasculature, thereby promoting distal metastasis (20). Activated platelets also release large amounts of platelet-derived growth factor (PDGF) (21), transforming growth factor-β (TGF-β), and vascular endothelial growth factor (VEGF) (22), strongly stimulating tumor cell proliferation, epithelial-mesenchymal transition, and fibrosis, and promoting tumor angiogenesis. This provides blood supply for micrometastases, allowing them to develop into clinically detectable metastatic tumors. In addition, a reduction in lymphocytes impairs the ability to effectively eliminate potential minimal residual cancer cells or circulating tumor cells postoperatively, leading to decreased immune surveillance, which is consistent with the mechanism described for NLR.

The core mechanism by which elevated MLR is associated with increased mortality lies in the fact that high MLR reflects myeloid-mediated immunosuppression and tissue repair, as well as tumor-promoting behavior. Monocytes can differentiate into M2-type macrophages. In the tumor microenvironment, most macrophages exhibit an M2 phenotype. M2 macrophages do not attack tumors; rather, they support tumor cell survival and dissemination by secreting anti-inflammatory factors, promoting angiogenesis and tissue repair (23). This may occur through secretion of cytokines such as IL-10 and TGF-β, which inhibit cytotoxic T cell and NK cell function, helping tumor cells evade immune attack; secretion of large amounts of pro-angiogenic factors such as VEGF and PDGF to supply nutrients and oxygen to tumors; and secretion of matrix metalloproteinases (MMPs) to degrade the extracellular matrix, facilitating tumor invasion and metastasis (24). Monocytes also play a mediating role in chronic inflammatory responses. This chronic inflammatory state generates reactive oxygen species (ROS), causing DNA damage that may promote tumorigenesis, and activates a series of signaling pathways (such as NF-κB and STAT3), promoting tumor cell survival and proliferation while resisting apoptosis. The mechanism of lymphocyte reduction is entirely consistent with that of NLR and PLR, as the immune system is unable to effectively eliminate residual or newly formed tumor cells postoperatively.

The superior predictive ability of PLR compared with NLR and MLR may be due to its direct association with the role of platelets in tumor biology. Platelets can first envelop circulating tumor cells, providing physical protection, and second, secrete growth factors such as platelet-derived growth factor (PDGF), directly promoting tumor progression (25). Lymphopenia, on the other hand, reflects a decreased anti-tumor immune capacity. NLR is influenced by factors such as infection and acute stress and mainly reflects the balance between neutrophils and lymphocytes (26), emphasizing inflammatory response rather than tumor-specific processes. MLR primarily reflects monocyte-related immune status, whose variation may be less stable and detectable than platelet counts, and is similarly more affected by inflammation and chronic diseases.

The innovation of this study lies in the analysis of interaction effects. The results indicate that NLR exhibits a synergistic effect with tumors located in the proximal esophagus. This may be because proximal esophageal tumors have a richer blood supply and are more prone to local lymph node or distant metastasis. Tumors in this region may trigger more pronounced local immune and inflammatory responses, leading to elevated NLR. In addition, the biological behavior of proximal esophageal tumors may be more aggressive, and higher NLR levels may further increase the risk of poor prognosis by promoting immune evasion and exacerbating the inflammatory microenvironment. NLR also shows a significant synergistic effect with infiltration depth. Greater tumor infiltration implies that tumor cells have spread to deeper tissue layers and may disrupt local vascular and neural structures, causing more pronounced local immune and inflammatory responses that promote neutrophil recruitment and activation, thereby elevating NLR levels. At the same time, deeply infiltrating tumors may be associated with more immune evasion phenomena, such as suppression of T cells or promotion of immunosuppressive cells (e.g., M2 macrophages) (27), further amplifying NLR, creating a vicious cycle, and increasing the risk of distant metastasis and mortality. PLR exhibits a significant synergistic effect with tumor volume. Larger tumors are often accompanied by tissue hypoxia, angiogenesis, and micro-bleeding during growth, which stimulate platelet aggregation and the release of pro-inflammatory factors. Platelets not only participate in hemostasis and tissue repair, but can also promote tumor angiogenesis, cell proliferation, and invasion through the secretion of VEGF, PDGF, and other factors, while protecting circulating tumor cells from immune clearance. These processes reinforce each other. Therefore, the synergistic effect between PLR and tumor volume may reflect platelet-mediated pro-inflammatory and immunosuppressive effects in large tumors. In patients with positive resection margins, the correlation between PLR and mortality risk is stronger. This may be because a positive margin indicates the presence of residual tumor or micrometastatic cells after surgery, which easily triggers local inflammatory responses (28). Platelets in this context may further enhance tumor invasiveness by promoting tumor cell growth and protecting circulating tumor cells from immune clearance, thereby increasing the risk of death. MLR also shows a synergistic effect with tumors located in the proximal esophagus, which is similarly related to the anatomical features of this region. The dominance of elevated monocytes reflected by MLR may be amplified in the context of proximal esophageal tumors, and the resulting inflammation-immune imbalance may more readily promote the survival, dissemination, and metastasis of residual or micrometastatic tumor cells, thereby increasing mortality risk.

Simultaneously, we also established threshold ranges for NLR, PLR, and MLR, which provide clinicians with a simple and effective tool for risk assessment. Specifically, when these indices exceed the clinically determined thresholds, the patients’ risk of death significantly increases, indicating that clinicians should implement closer monitoring during postoperative follow-up for such patients. By utilizing these threshold ranges, physicians can more accurately identify high-risk patients, adjust treatment plans in a timely manner, or increase monitoring frequency, thereby improving long-term prognosis.

By integrating our findings with previous studies, we constructed a risk score that combines inflammatory status with key clinical features, allowing for more accurate identification of truly high-risk patients. Clinicians can use this information to tailor individualized follow-up and management plans. For low-risk patients, unnecessary examinations could be reduced to avoid wasting medical resources. However, it should be noted that the risk score we developed is exploratory, and its practical application is currently limited. Future studies may build on our work to develop more precise predictive models.

This study also has certain limitations. First, it is a retrospective study, which may introduce selection bias in data collection. Second, it is a single-center study with a relatively small sample size, and the baseline analysis did not fully account for other potential influencing factors. Although the multivariate Cox analysis adhered to the events per variable (EPV) principle and the overall stability of the model is acceptable, the limited number of death events reduces the precision of the model, leading to wider confidence intervals. Therefore, these findings should be interpreted with caution, and larger or multicenter studies are needed to further validate the reliability of these associations in the future. This study collected peripheral blood inflammatory markers only at the time of discharge, which may not fully capture the dynamic changes in the systemic inflammatory response. Future studies could incorporate multiple timepoints, such as one month and three months after discharge, to more comprehensively evaluate the temporal variation of inflammatory levels and their association with prognosis. This study performed multiple Cox analyses, which introduces a risk of false-positive findings due to multiple comparisons. After false discovery rate (FDR) correction, some borderline significant interaction effects became non-significant, indicating that the conclusions from the interaction analyses are exploratory and require further validation in studies with larger sample sizes.

## 5 Conclusion

This study demonstrates that NLR, PLR, and MLR are all significantly positively associated, in a linear or near-linear manner, with the three-year risk of death in patients with early-stage esophageal cancer undergoing ESD. NLR shows significant synergistic effects with tumors located in the proximal esophagus and with invasion depth. PLR exhibits significant synergistic effects with tumor volume and resection margin status. MLR also shows significant synergistic effects with tumors located in the proximal esophagus. When these indices exceed specific thresholds, the risk of patient death increases markedly, indicating that they can serve as early warning markers for high-risk postoperative patients. This study provides new biomarker evidence for prognostic management in patients with early-stage esophageal cancer.

## Data Availability

The original contributions presented in the study are included in the article/**Supplementary Material**. Further inquiries can be directed to the corresponding author.

## Supplementary material

Supplementary Figure 1 Patient selection flowchart.

Supplementary Figure 2 Graphical abstract.
