# Supplementary figures and images for: Predictive value of neutrophil-to-lymphocyte ratio, platelet-to-lymphocyte ratio, and monocyte-to-lymphocyte ratio for three-year survival in patients with early esophageal cancer undergoing endoscopic submucosal dissection
Source: Front Oncol. 2026 Jan 7;15:1714984. doi: 10.3389/fonc.2025.1714984 (PMC12819809; doi:10.3389/fonc.2025.1714984)

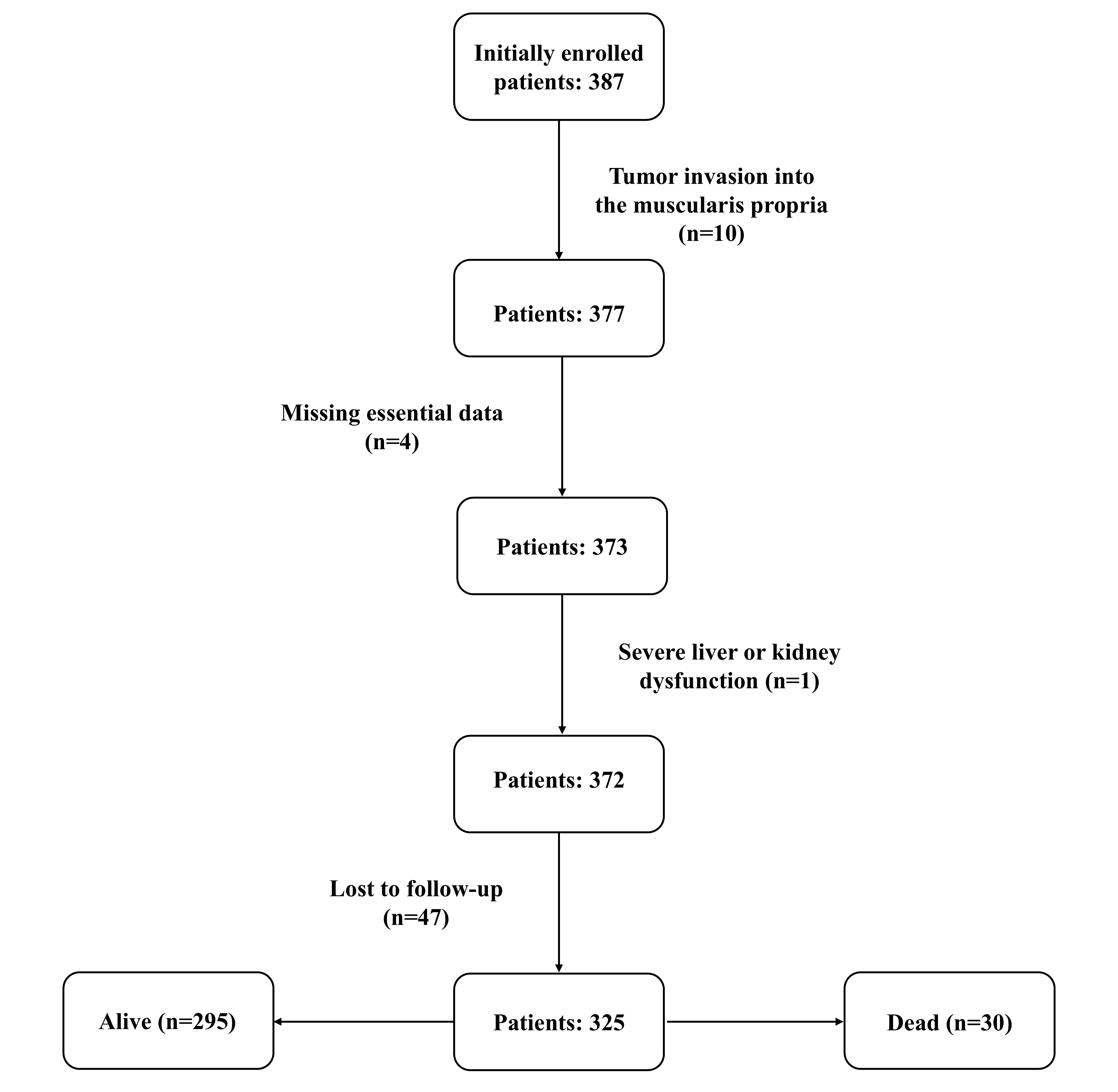

Supplement: Supplementary Figure 1 — Patient selection flowchart. [file Image1.tif]

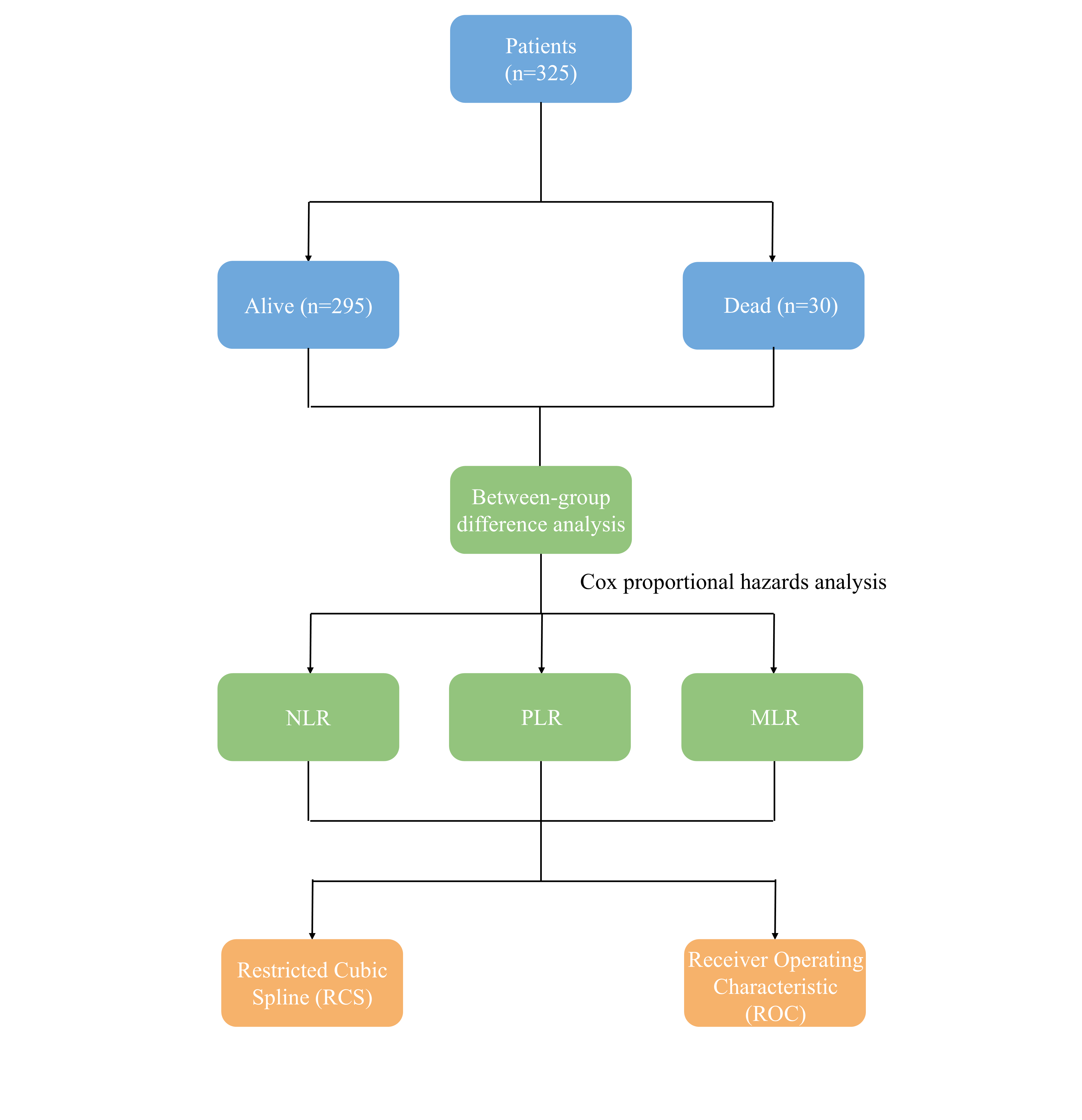

Supplement: Supplementary Figure 2 — Graphical abstract. [file Image2.tif]
