# Supplementary material for: Predictive value of neutrophil-to-lymphocyte ratio, platelet-to-lymphocyte ratio, and monocyte-to-lymphocyte ratio for three-year survival in patients with early esophageal cancer undergoing endoscopic submucosal dissection
Source: Front Oncol. 2026 Jan 7;15:1714984. doi: 10.3389/fonc.2025.1714984 (PMC12819809; doi:10.3389/fonc.2025.1714984)
Supplement: Supplementary file 3 [file Table1.docx]

| **Supplementary Table. 1 Multivariate Cox regression analysis adjusted for potential confounding factors** | | | | | | | |
| --- | --- | --- | --- | --- | --- | --- | --- |
| **Term** | **Coef** | **Std error** | **statistic** | **P value** | **HR** | **CI-lower** | **CI-upper** |
| **NLR** | 0.267 | 0.072 | 3.732 | 0.000 | 1.306 | 1.135 | 1.503 |
| **PLR** | 0.005 | 0.001 | 5.242 | 0.000 | 1.005 | 1.003 | 1.007 |
| **MLR** | 1.172 | 0.411 | 2.852 | 0.004 | 3.228 | 1.443 | 7.223 |
| **Hypertension** | 0.338 | 0.135 | 2.499 | 0.012 | 1.402 | 1.076 | 1.828 |
| **Lesion.location** | -0.049 | 0.012 | -3.939 | 0.000 | 0.952 | 0.929 | 0.976 |
| **Tumor.Size** | 0.325 | 0.094 | 3.444 | 0.001 | 1.384 | 1.150 | 1.664 |
| **Resection** | 0.612 | 0.144 | 4.256 | 0.000 | 1.843 | 1.391 | 2.443 |
| **Differentiation** | 0.510 | 0.089 | 5.694 | 0.000 | 1.665 | 1.397 | 1.984 |
| **Invasion** | 0.796 | 0.148 | 5.369 | 0.000 | 2.218 | 1.658 | 2.966 |
| **lymphovascular.invasion** | 0.384 | 0.144 | 2.658 | 0.008 | 1.468 | 1.106 | 1.949 |
| **LOS** | 0.057 | 0.022 | 2.644 | 0.008 | 1.059 | 1.015 | 1.105 |
| **Surgery.Time** | 0.011 | 0.003 | 3.749 | 0.000 | 1.011 | 1.005 | 1.017 |
| **Age** | -0.004 | 0.007 | -0.517 | 0.605 | 0.996 | 0.982 | 1.011 |
| **BMI** | -0.018 | 0.020 | -0.864 | 0.387 | 0.982 | 0.944 | 1.023 |
| **Gender** | 0.283 | 0.186 | 1.523 | 0.128 | 1.327 | 0.922 | 1.909 |
| **Adjuvant.therapy** | -0.074 | 0.226 | -0.329 | 0.742 | 0.928 | 0.596 | 1.446 |
