# Supplementary material for: Predictive value of neutrophil-to-lymphocyte ratio, platelet-to-lymphocyte ratio, and monocyte-to-lymphocyte ratio for three-year survival in patients with early esophageal cancer undergoing endoscopic submucosal dissection
Source: Front Oncol. 2026 Jan 7;15:1714984. doi: 10.3389/fonc.2025.1714984 (PMC12819809; doi:10.3389/fonc.2025.1714984)
Supplement: Supplementary file 4 [file Table2.docx]

| **Supplementary Table. 2 False Discovery Rate (FDR)-Corrected Interaction Analyses and Proportional Hazards Assumption Tests for NLR, PLR, and MLR** | | |
| --- | --- | --- |
| **Term** | **adj.P (FDR)** | **GLOBAL P** |
| **NLR** |  | 0.270 |
| **NLR.GroupQ1** |  | 0.120 |
| **NLR.GroupQ2** |  |  |
| **NLR.GroupQ3** |  |  |
| **NLR.GroupQ4** |  |  |
| **NLR*Hypertension** | 0.467 | 0.580 |
| **NLR*Lesion location** | 0.077 | 0.460 |
| **NLR*Tumor Size** | 0.463 | 0.560 |
| **NLR*Resection** | 0.986 | 0.280 |
| **NLR*Differentiation** | 0.210 | 0.169 |
| **NLR*Invasion** | 0.077 | 0.640 |
| **NLR*lymphovascular invasion** | 0.986 | 0.370 |
| **NLR*LOS** | 0.463 | 0.290 |
| **NLR*Surgery Time** | 0.467 | 0.056 |
|  |  |  |
| **PLR** |  | 0.160 |
| **PLR.GroupQ1** |  | 0.130 |
| **PLR.GroupQ2** |  |  |
| **PLR.GroupQ3** |  |  |
| **PLR.GroupQ4** |  |  |
| **PLR*Hypertension** | 0.783 | 0.520 |
| **PLR*Lesion location** | 0.783 | 0.220 |
| **PLR*Tumor Size** | 0.117 | 0.300 |
| **PLR*Resection** | 0.194 | 0.460 |
| **PLR*Differentiation** | 0.455 | 0.244 |
| **PLR*Invasion** | 0.818 | 0.530 |
| **PLR*lymphovascular invasion** | 0.455 | 0.370 |
| **PLR*LOS** | 0.783 | 0.500 |
| **PLR*Surgery Time** | 0.783 | 0.085 |
|  |  |  |
| **MLR** |  | 0.140 |
| **MLR.GroupQ1** |  | 0.200 |
| **MLR.GroupQ2** |  |  |
| **MLR.GroupQ3** |  |  |
| **MLR.GroupQ4** |  |  |
| **MLR*Hypertension** | 0.867 | 0.620 |
| **MLR*Lesion location** | 0.054 | 0.400 |
| **MLR*Tumor Size** | 0.867 | 0.590 |
| **MLR*Resection** | 0.867 | 0.210 |
| **MLR*differentiation** | 0.917 | 0.480 |
| **MLR*Invasion** | 0.867 | 0.080 |
| **MLR*lymphovascular invasion** | 0.956 | 0.410 |
| **MLR*LOS** | 0.917 | 0.510 |
| **MLR*Surgery Time** | 0.867 | 0.091 |
